# Role of the Zinc Finger Transcription Factor SltA in Morphogenesis and Sterigmatocystin Biosynthesis in the Fungus *Aspergillus nidulans*

**DOI:** 10.1371/journal.pone.0068492

**Published:** 2013-07-01

**Authors:** Sourabha Shantappa, Sourabh Dhingra, Patricia Hernández-Ortiz, Eduardo A. Espeso, Ana M. Calvo

**Affiliations:** 1 Department of Biological Sciences, Northern Illinois University, DeKalb, Illinois, United States of America; 2 CSIC (Consejo Superior de Investigaciones Cientificas), Centro Investigaciones Biológicas, Department of Cellular and Molecular Biology, Madrid, Spain; Universidade de Sao Paulo, Brazil

## Abstract

Potassium, a widely accepted macronutrient, is vital for many physiological processes such as regulation of cell volume, maintenance of intracellular pH, synthesis of proteins and activation of enzymes in filamentous fungi. Another cation, calcium, plays an essential role in many signaling processes from lower to higher eukaryotes. Imbalance in the intracellular ionic levels of potassium or calcium causes adverse effects on cell growth, morphology and development, and eventually death. Previous studies on the adaptation of *Aspergillus nidulans* to salt and osmotic stress conditions have revealed the role of SltA, a C_2_H_2_ zinc finger transcription factor in cation homeostasis. SltA is highly conserved in the Ascomycota phylum with no identifiable homolog in *S. cerevisiae* and other yeast-like fungi, and prevents toxicity by the cations Na^+^, K^+^, Li^+^, Cs^+^ and Mg^2+^, but not by Ca^2+^. However its role in morphology and biosynthesis of natural products such as mycotoxins remained unknown. This study shows the first characterization of the role of calcium and SltA fungal homologs in morphogenesis using the model system *A. nidulans*. Addition of potassium to *sltA* deletion mutants resulted in decreased levels of sterigmatocystin production. A similar phenotype was observed for both types of mutants in *veA1* and *veA* ^+^ genetic background. Expression of the sterigmatocystin genes *aflR* and *stcU* was strongly reduced in *sltA* deletion mutant when K^+^ was added. Additionally, increased concentrations of K^+^ drastically reduced sexual and asexual development, as well as radial growth in deletion *sltA* colonies. This reduction was accompanied by lower expression of the morphology related genes *nsdD*, *steA* and *brlA*. Interestingly, addition of calcium was able to stimulate asexual and sexual development and remediate the deletion *sltA* phenotype, including defects in morphology and toxin production.

## Introduction

Filamentous fungi inhabit a wide range of both indoor and outdoor environments, which includes soil, plants, animals and human hosts. They are also capable of growing in extreme environmental niches such as salterns, hot springs, deserts, deep sea sediments, bird excreta, aquatic habitats, mine drainages and in the crevices of rocks. Every single environmental condition mentioned above presents various forms of stresses including osmotic stress, oxidative stress, nutrient deprivation, changes in pH and heat shock. Fungi have developed sophisticated mechanisms to alleviate the extracellular stress associated with these harsh conditions and thereby promote growth and survival.

Potassium is a very important nutrient required for many physiological processes such as regulation of cell volume, maintenance of intracellular pH, synthesis of proteins and activation of enzymes in several organisms such as plants, animals, bacteria and filamentous fungi. However, under hypertonic ambient conditions, high-salt concentrations cause cells to lose water when cations enter the cell. Na^+^ is highly toxic and is usually maintained in low levels, while K^+^ is accumulated at high concentrations in the cell. When the intracellular concentration of Na^+^ approaches that of K^+^, the cell growth is inhibited. Thus, in order to maintain cation homeostasis, specific transporters facilitate accumulation of K^+^ in the cytoplasm and extrusion of Na^+^ out of the cells. For more than five decades, yeast cells have been employed as model systems to study the alkali metal cation transport and homeostasis [1].

In *A. nidulans* the activity of a zinc-finger transcription factor, SltA, has been associated with tolerance to salt stress. SltA, also called StzA in some reports, was identified in early studies of salt tolerance in *A. nidulans* [2]. The *sltA* gene, located on chromosome VI in *A. nidulans* encodes a 698-amino acid-residue protein containing three classical Cys_2_His_2_ zinc fingers [3,4]. Phylogenetic distribution of this transcription factor is limited to the Pezyzomycotina phylum, subsequently with no identifiable homolog in *S. cerevisiae* [4,5]. O’Mahony et al., (2002) [6] showed that a *sltA1* mutant was defective in arginase activity, which also coincided with reduced salt tolerance in *A. nidulans*. Studies by O’Neil et al., 2002 [3] reported that besides exhibiting tolerance to salt stress, SltA also has an important role in DNA repair functions. The null allele of *sltA* causes sensitivity to elevated concentrations of monovalent cations such as sodium, lithium, rubidium and potassium, among others, and to divalent cations such as manganese and magnesium, but not to calcium [4]. Another important phenotype is the sensitivity of the null *sltA* strain to alkalinity, shared with null alleles of other general transcription factors such as CrzA [4], the regulator of calcium stress, and PacC, the well-known mediator of ambient pH regulation in *A. nidulans* [7,8]. ACE1 from *Trichoderma* *reseii* , a transcription factor with strong similarity to SltA that regulates expression of cellulase and xylanase promoters, binds to the consensus 5'-AGGCA-3' [9]. SltA also binds to this target, which has been found in genes under its regulation such as the vacuolar calcium exchanger, *vcxA* [4,10].

Despite this extensive work on the regulation of cation tolerance by SltA, nothing was known about its possible role in the regulation of fungal morphogenesis and secondary metabolism. *Aspergillus nidulans* efficiently disseminates by developing asexually formed air-borne conidia on specialized structures denominated conidiophores. Production of these structures is spatially and temporarily regulated [11,12]. Additionally, *A. nidulans* also develops fruiting bodies called cleistothecia where sexual spores, or ascospores, are formed [13]. Previous studies have revealed genetic regulatory links governing both morphogenesis and secondary metabolism in filamentous fungi [14]. For example, we have previously shown that the conserved global regulatory gene *veA*, initially known to control sexual and asexual development in *A. nidulans* [15], was also required for the synthesis of numerous secondary metabolites, including sterigmatocystin (ST) [16,17]. ST is a mycotoxin structurally similar to aflatoxins (AF), highly carcinogenic compounds produced by related *Aspergillus* species including *A. flavus, A. parasiticus*, and *A* *. nomiusi* [18–21]. Because both ST and AF are synthesized through a conserved biosynthetic pathway where ST is the penultimate precursor, and due to the fact that regulatory mechanisms controlling the production of both toxins tend to be conserved, *A. nidulans* has been used as an efficient model system in the study of AF/ST genetic regulation [22–26]. It is possible that elements involved in the regulation of cation homeostasis could also influence both morphogenesis and mycotoxin biosynthesis. In this study we showed that indeed *sltA*, in addition to its described role in cation stress tolerance, is also necessary for morphological development and sterigmatocystin production in *A. nidulans*.

## Materials and Methods

### Strains and culture conditions

The *Aspergillus nidulans* strains used in this study are listed in Table 1. Meiotic recombination was performed in order to obtain the null *sltA* mutant in a *veA* ^+^ background from the original ∆*sltA* mutant carrying the *veA1* allele. The ∆*sltA, veA1* strain was initially obtained as previously described [4]. Essentially, the *sltA* coding region was precisely deleted by replacement with the *Aspergillus fumigatus riboB* gene. All strains generated by Spielvogel et al., 2008 [4] contained the *veA1* mutation. Genetic techniques were performed as previously described [27]. MAD1685 (*inoB2*, ∆*sltA*::*riboB*, *veA1*) was crossed with WIM126 (*pabaA1*, *yA2*, *veA* ^+^) and the resulting progenies were screened for *veA* ^+^ genotype and confirmed by sequencing.

**Table 1:** *A. nidulans* strains used in this study.

**Strain**	**Genotype**	**Source/ Reference**
HHF27A	*veA1*	Findon et al., 2010
HHF27B	∆*sltA::riboB*_*Af*_ *, veA1*	Findon et al., 2010
FGSC4	*veA* ^*+*^	Fungal Genetics Stock Center
MAD1685	*inoB2*, ∆*sltA::riboB*_*Af*_ *, veA1*	This study
WIM126	*pabaA1,yA2,veA* ^*+*^	Fungal Genetics Stock Center
RNKT 5.1	*pyroA4, veA* ^*+*^	This study
RSS1.6P	∆*sltA::riboB*_*Af*_ *, veA* ^+^	This study
RSS1.6P-com	*veA* ^+^, ∆*sltA::riboB*_*Af*_ *, / sltA*	This study

Strains were grown on solid glucose minimal medium, GMM [28], at 37^o^C unless otherwise indicated. Agar (10 g/liter) was added for solid medium. Strains were stored as glycerol stocks at -80^o^C.

### Fungal growth and developmental studies

SltA deletion strains (with *veA1* or *veA* ^+^ genetic background, HHF27B and RSS1.6P respectively) along with the corresponding prototrophic controls (HHF27A and FGSC4 respectively) were point inoculated on GMM, and GMM, supplemented with 200 mM or 400 mM KCl, unless otherwise indicated. Cultures were then incubated for 5 days at 37°C. Fungal growth was estimated as colony diameter.

Conidiation was examined on plates containing 25 ml of solid GMM and GMM, supplemented with 200 mM or 400 mM KCl that were top-agar inoculated with 5 ml of GMM, GMM supplemented with 200 mM or 400 mM KCl containing 5 x10^6^ spores from the strains ∆*sltA, veA1* (HHF27B) or ∆*sltA, veA* ^+^ (RSS1.6P), or from the corresponding control strains. The cultures were incubated for 5 days at 37°C. The experiment was carried out with three replicates. After 5 days, 8-mm-diameter cores were obtained from each spread plate and homogenized in water. Conidial production was quantified using a hemocytometer. For evaluation of cleistothecial production, wrapped top-agar inoculated cultures were incubated for 10 days at 37°C, when 16-mm-diameter cores were obtained from each culture and sprayed with 70% ethanol to enhance visualization of cleistothecia.

Alternatively, 10^7^ spores/mL of the ∆*sltA, veA* ^+^ (RSS1.6P) strain, along with its control, were inoculated into flasks containing either GMM, GMM supplemented with 200 mM KCl or GMM supplemented with 10 mM CaCl_2_. Cultures were grown in a shaker incubator for 24 hours at 37°C at 250 rpm. Then, approximately 2 grams of mycelia were transferred onto each of the corresponding solid plates containing GMM or GMM supplemented with salts (KCl or CaCl_2_). The plates were then incubated for additional 24 and 48 hours. Then, 8-mm-diameter cores were obtained from each plate and homogenized. Conidial and Hülle cell production were evaluated using a hemocytometer. The experiment was carried out with three replicates.

### Mycotoxin analysis

Three cores (16-mm diameter) were collected from each replicate of the cultures described above (both spread plating and shift culture) and extracted with CHCl_3_. The extracts were analyzed by thin-layer chromatography (TLC). Benzene: glacial acetic acid (95:5 [vol/vol]) or alternatively toluene: ethyl acetate: acetic acid (80:10:10 [vol/vol/vol]) were used as solvent systems. The silica gel TLC plates were then sprayed with aluminum chloride (12.5% concentration in ethanol 95%) and baked for 10 min at 80°C prior to viewing to intensify ST fluorescence upon exposure to long-wave (375 nm) UV light. ST purchased from Sigma was used as a standard. Densitometry of the TLC images was carried out using Scion Image 4.03 software.

### Gene expression analysis

Expression analysis of developmental genes was carried out using mycelia harvested from shift (liquid to solid) cultures of ∆*sltA* (RSS1.6P) and its *veA* ^+^ control strain (FGSC4) at 24 and 48 hours after the shift. Samples were frozen and stored at -80°C. Expression analysis of mycotoxin genes was performed from mycelia harvested from liquid shaken cultures. In both cases, samples were lyophilized, followed by RNA isolation using Trizol® reagent according to the manufacturer recommendations. Gene expression was analyzed by quantitative reverse transcription-PCR (qRT-PCR). Five micrograms of total RNA were treated with RQ1 RNAse-Free DNAse (Promega) and reverse transcribed with Moloney murine leukemia virus (MMLV) reverse transcriptase (Promega) to synthesize cDNA. Applied Biosystems 7000 Real-Time PCR System was used for qRT-PCR and SYBR green dye was used for fluorescence detection. The primer pairs used for qRT-PCR are listed in Table 2. The data for each gene were normalized to the *A. nidulans* housekeeping gene, *actin*-F and the relative expression levels were calculated using the 2^-∆CT^ method described previously [29].

**Table 2:** List of primers used in this study.

**Primer name**	** Sequence (5’–3’)**
*actin*-F	ATGGAAGAGGAAGTTGCTGCTCTCGTTATCGACAATGGTTC
*actin*-R	CAATGGAGGGGAAGACGGCACGGG
*nsdD*-F	CATCTCACCAGCCACAATTACAGGCGGAACCATCAC
*nsdD*-R	TTGCGAGCCAGACACAGAGGTCATAACAGTGCTTGC
*steA*-F	TCCAGCAAATGGAACCGTGGAATCAGGTGCTC
*steA*-R	GAAGGGATGGGGCAAGAATGAGACTTCTGCGGGTAA
*brlA-*F	AGCTGCCTGGTGACGGTAGTTGTTGTTGGTGTTGC
*brlA-*R	CAGGAACGAATGCCTATGCCCGACTTTCTCTCTGGA
*aflR*-F	ATGGAGCCCCCAGCGATCAGCCAG
*aflR*-R	TTGGTGATGGTGCTGTCTTTGGCTGCTCAAC
*stcU*-F	GTCTCGATGGAAAAGTCGCTCTGGTAACTGGGG
*stcU*-R	CATGCCCGAACGAGACAATTCCCGCGTTAG
sltA39	GAGACCACCAGGGCCGG
sltA47	AGGGCTCCGCAACTTAGACTCG
sltA13	CTTCCGTTGGATTTGGTCACG
sltA_F	CTGTCCCGTCCTCTCCTCACC
sltA_R	GGTACAATCCCGTCATGC

Expression profile of *sltA* during vegetative growth, as well as during asexual and sexual-developmental phases was examined in a wild-type strain (*veA* ^+^, WIM126) grown for 12, 18 and 24 h in liquid GMM to obtain vegetative mycelial growth. For induction of asexual or sexual development, vegetative mycelia were collected from liquid GMM, placed on top of solid GMM and incubated in the light (conducive to asexual development) or in the dark (conducive to sexual development). Samples were harvested at 6, 12, 24 and 48 h after the shift. Total RNA was extracted with Trizol® and analyzed by Northern blot. Extracted RNAs were probed with a radioactive-labeled *sltA* coding region PCR amplified from *A. nidulans* genomic DNA with primers SltA39 and SltA13 (Table 2).

### In silico analysis of the promoter region

In order to identify putative SltA transcription factor binding sites, approximately 3.5 kb sequences upstream of the coding region of *nsdD*, *brlA*, *steA* and *aflR* were obtained from the NCBI database (http://www.ncbi.nlm.nih.gov/) and the *Aspergillus* Comparative Database (http://www.broadinstitute.org/annotation/genome/aspergillus_group/MultiHome.html) for *A. nidulans*. The putative SltA binding sites were identified by comparison with the consensus sequences (5’ AGGCA 3’) previously described [4,5].

## Results

### *sltA* is important for growth and asexual development in *A. nidulans*

**Figure 1:**
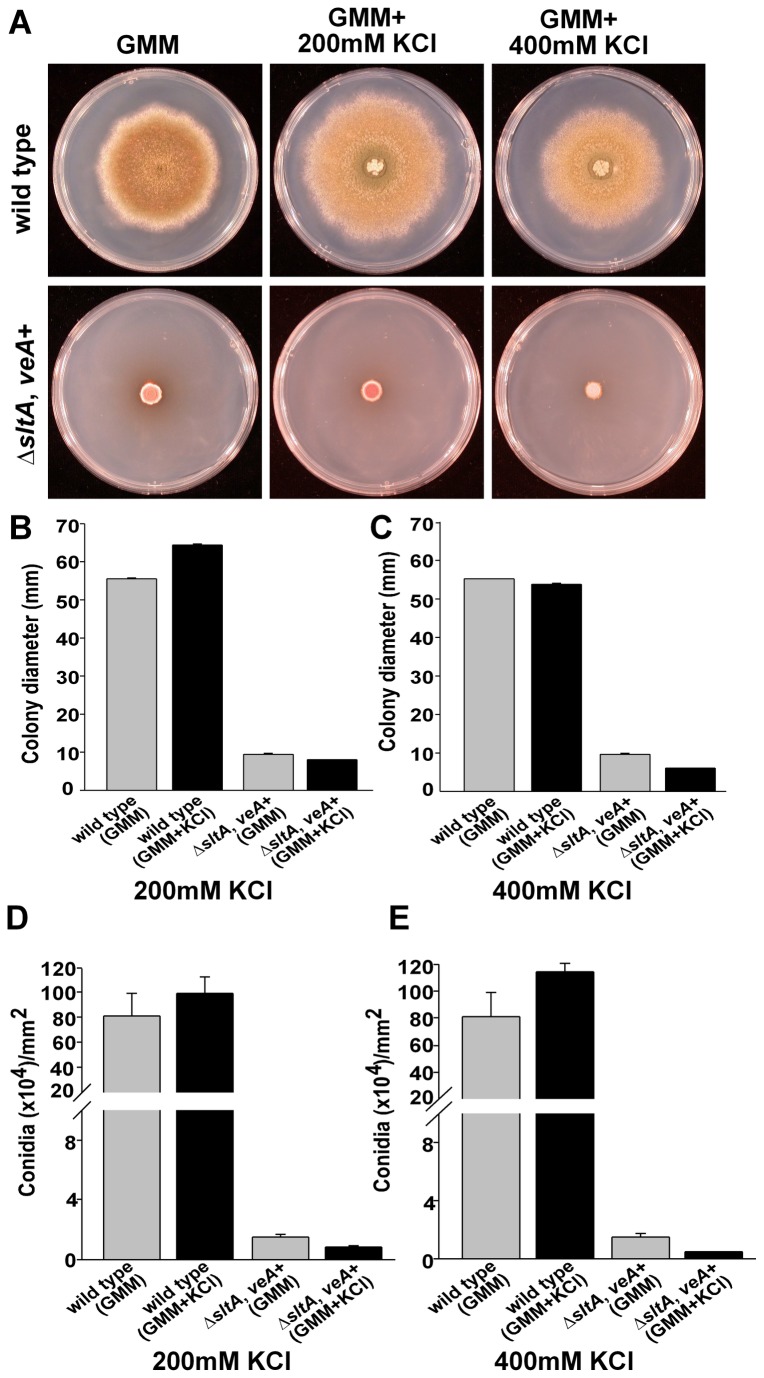
SltA is necessary for normal growth and conidiation. A) Wild-type (FGSC4) and ∆*sltA*, *veA* ^*+*^ (RSS1.6P) point-inoculated cultures containing GMM, or GMM supplemented with 200 mM or 400 mM KCl were incubated at 37°C for 5 days. B) & C) Measurement of the radial colony growth. D) & E) Quantification of conidial production from top-agar inoculated cultures (5 x 10^6^ spores/plate). Values are means of three replicates. The error bar indicates standard error.

**Figure 2:**
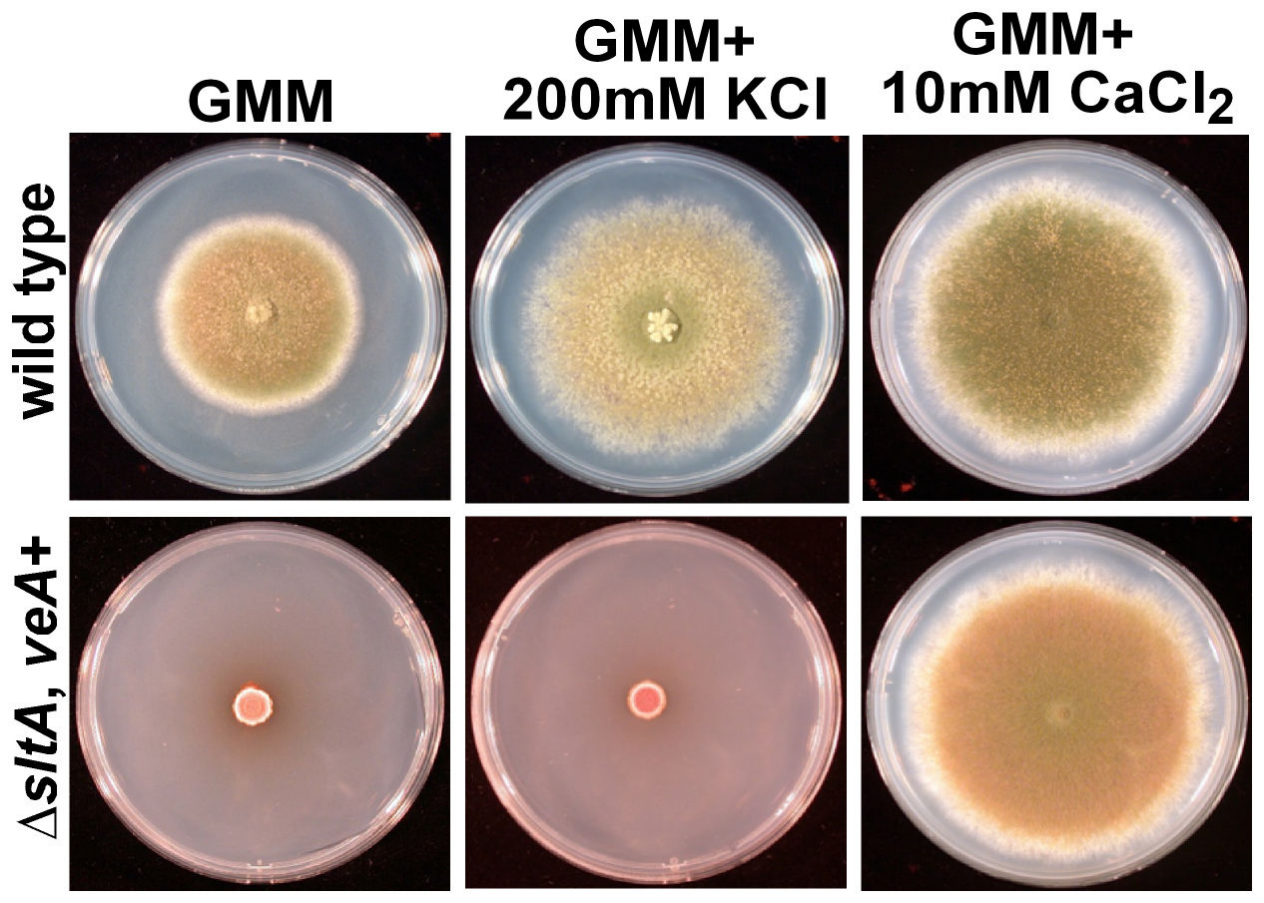
Calcium remediated the growth defect in the *sltA* deletion mutant. Photographs of point-inoculated colonies of *A. nidulans* ∆*sltA, veA* ^+^ (RSS1.6P) and its control strain (FGSC4). The strains were grown on GMM, GMM supplemented with 200 mM KCl, or GMM supplemented with 10 mM CaCl_2_. The plates were incubated for 5 days at 37°C.

Classically Käfer’s GMM containing sodium nitrate as main nitrogen source has been used to study regulation of mycotoxin production and many aspects of development in *Aspergillus nidulans* [16,25,26,30–32]. For this reason this medium was used to investigate the role of SltA transcription factor in these processes. In previous studies carried out by Spielvogel et al., 2008 [4] on *sltA* mutant in *veA1* genetic background only a slight reduction of growth was observed on Cove’s minimal medium with ammonium tartrate as nitrogen source (not conducive to mycotoxin production in *A. nidulans*) but strong sensitivity was detected to elevated concentrations of mono and divalent cations (Figure S1) with the exception of calcium [4]. In our current study, the *A. nidulans sltA* deletion strains and their control strains were exposed to different salt concentrations in GMM in order to elucidate the possible role of *sltA* on fungal growth and asexual development in relationship with the cation concentration in the environment, and specifically we selected K^+^ for this purpose. The growth of ∆*sltA* strains was significantly reduced in comparison with the control strain, and this reduction was further pronounced in the presence of K^+^ (Figures 1 and S1, S2), particularly in the presence of 400 mM KCl (Figure 1A and 1C Figures S2A and S2C), indicating that *sltA* is important for fungal growth. The reduction of growth observed on GMM was independent of the type of *veA* allele. Interestingly, the addition of 10 mM CaCl_2_ to GMM resulted in a significant increase in the fungal colony growth in the *sltA* deletion mutant strain (Figure 2).

In order to prevent the possible effect of growth reduction on the evaluation of the role of SltA on morphogenesis, the strains were top-agar inoculated, or alternatively, a liquid-to-solid mycelial transfer was implemented using the same amount of biomass for all strains. A decrease in the production of conidia was observed in the absence of *sltA* (in both, *veA1* and *veA* ^+^ genetic backgrounds) particularly in the presence of 200 mM KCl (Figures 1D and S2D) and even more as the concentration was increased to 400 mM (Figures 1E and S2E).

### *sltA* is required for normal *brlA* expression

**Figure 3:**
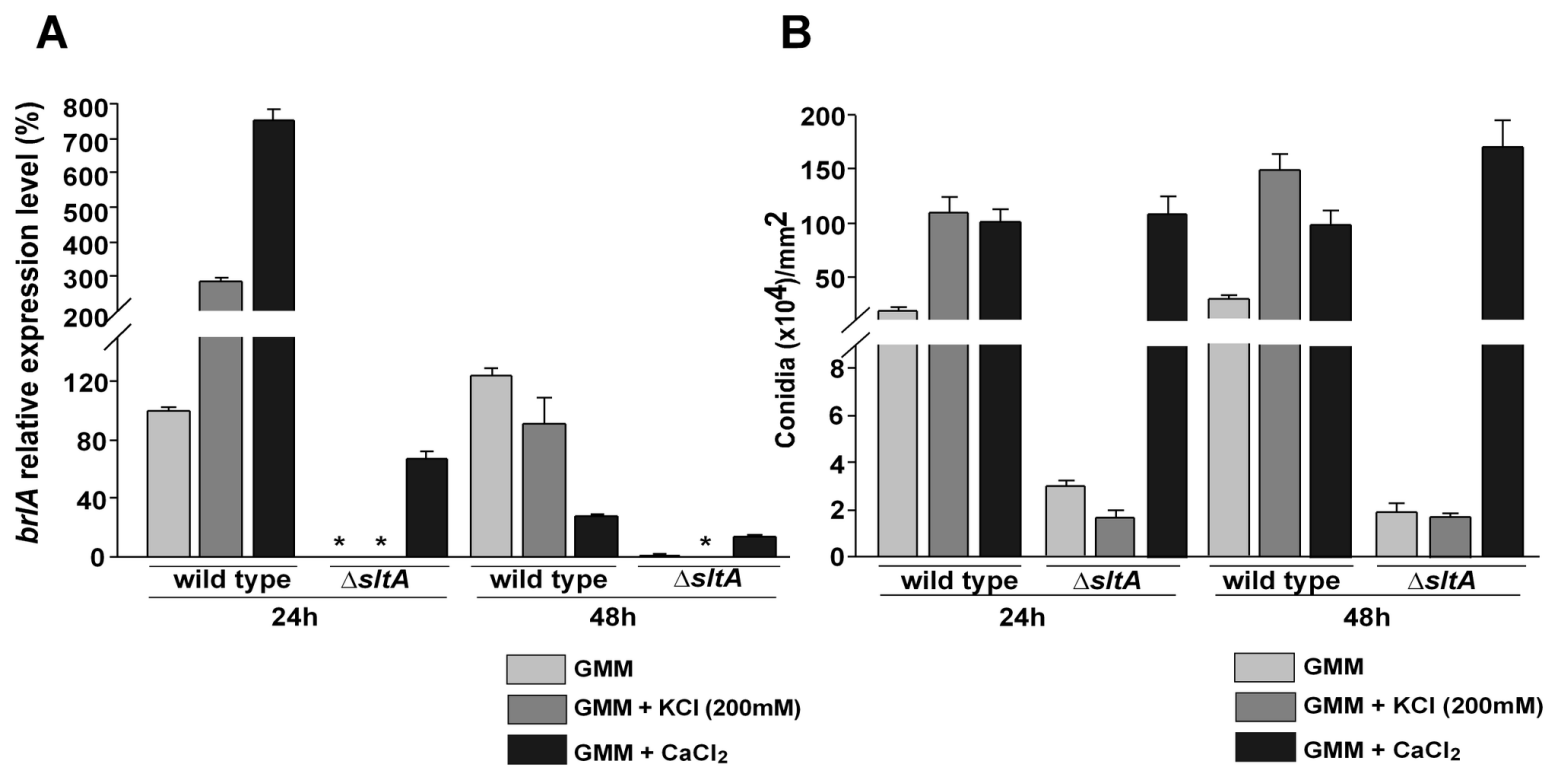
Expression of *brlA* is affected by SltA. A) *brlA* expression analysis by qRT-PCR. The values were normalized to the wild-type (grown on GMM -24 h) levels considered as 100. Strains were initially grown in liquid medium in a shaker incubator for 24 hours at 250 rpm. Then equal amounts of mycelia were homogeneously spread onto solid medium GMM, or GMM, supplemented with 200 mM KCl or with 10 mM CaCl_2_ (time point - 0 h) and further incubated at 37°C. Total RNA was extracted using Trizol® from mycelial samples collected at 24 and 48 hours after the shift. B) Quantification of conidia produced by ∆*sltA, veA* ^+^ (RSS1.6P) and its wild-type control (FGSC4) under the same experimental conditions. An 8-mm-diameter core was extracted from the cultures at 24 and 48 hours after the shift and homogenized in water. Spores were counted with a hemocytometer. Values are means of three replicates. The error bar indicates standard error. Asterisk indicates not detected.

Expression of *brlA*, a master gene encoding a transcription factor necessary for asexual development in *A. nidulans* [11], was analyzed in ∆*sltA, veA* ^+^ (RSS1.6P) and its control strain (FGSC4). In order to obtain quality RNA from mycelia, the strains were grown in liquid shaken culture for about 24 hours and then equal amounts of mycelia were transferred onto solid plates and incubated for an additional 24 and 48 hours to allow asexual development. Expression analysis of *brlA* was dependent on the cation content for the wild-type strain. At 24 hrs *brlA* mRNA levels were increased more than twice when K^+^ was added, and more than 7 times when Ca^2+^ was supplemented. This positive effect on *brlA* transcription resulted in an increase in conidiation in both K^+^ and Ca^2+^ supplemented medium (Figure 3A and 3B). Asexual development was greatly reduced in the *sltA* deletion mutant grown with or without K^+^ at 24 hour time point, accompanied by a decrease in *brlA* expression, as compared to the wild type (Figure 3A and 3B). Addition of 10 mM CaCl_2_ to GMM resulted in a significant recovery of *brlA* expression in the ∆*sltA* strain (Figure 3A), with an increase in conidiation (Figure 3B). This positive effect of calcium on *brlA* expression in both wild type and null *sltA* decreased over time (Figure 3A). This transitory effect was also observed at 24 h for the wild-type strain when K+ was supplemented to the medium.

### *sltA* is necessary for sexual development

**Figure 4:**
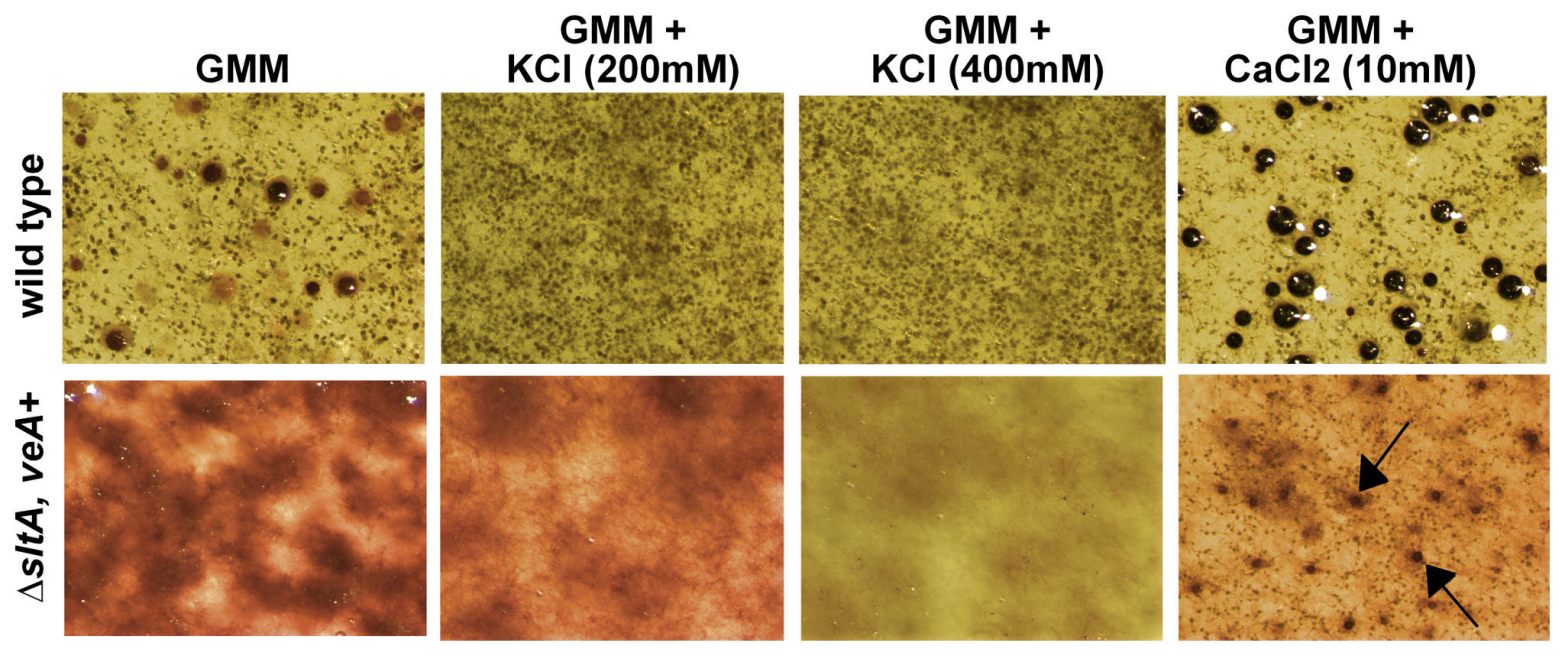
Effect of *sltA* deletion on *A. nidulans* cleistothecial production. Micrographs of fruiting bodies (cleistothecia) in ∆*sltA, veA* ^+^ (RSS1.6P) and wild type (FGSC4). Strains were top-inoculated with 5 x10^6^ conidia per plate on GMM, GMM supplemented with KCl (200 mM or 400 mM), or GMM supplemented with 10 mM CaCl_2_, and incubated at 37°C for 10 days. Plates were wrapped to promote sexual development. Cultures were sprayed with 70% ethanol to facilitate the visualization of cleistothecia. Arrows indicate cleistothecia in the ∆*sltA, veA* ^+^ culture. Magnification: x50.

Sexual development was also altered in the ∆*sltA, veA* ^+^ (RSS1.6P) strain. Deletion of *sltA* resulted in a strain unable to produce cleistothecia (Figure 4). In contrast to the positive effect of addition of K^+^ on asexual development, production of mature cleistothecia was negatively affected by the addition of this cation in the wild type (Figure 4). However, calcium promoted both asexual and sexual development, increasing cleistothecia formation in both *sltA* mutant and wild-type strains (Figure 4).

**Figure 5:**
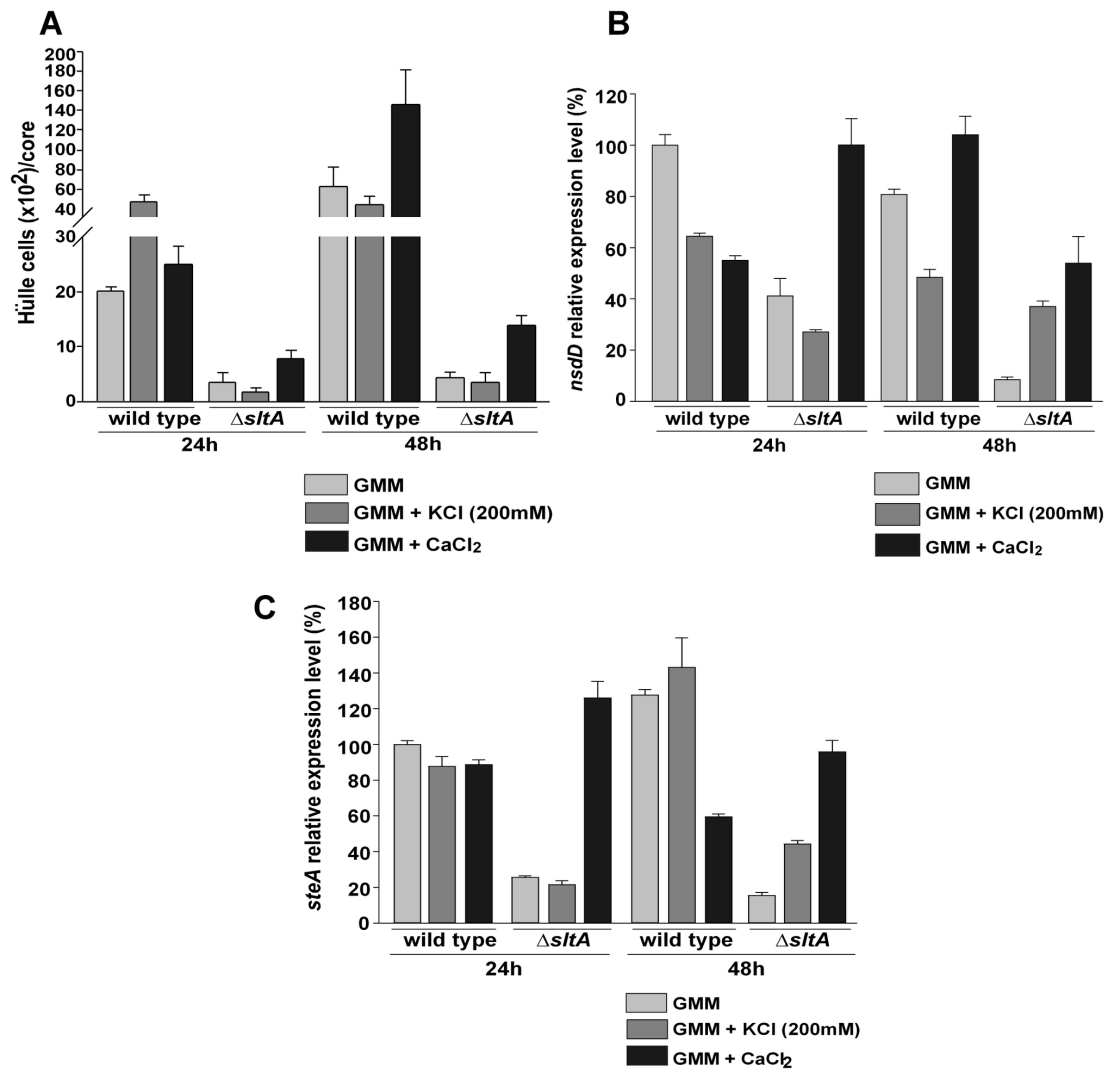
SltA is required for normal Hülle cell production and regulates *nsdD* and *steA* expression. A) Quantification of Hülle cells produced by wild-type (FGSC4) and ∆*sltA, veA* ^+^ strains (RSS1.6P). The strains were first grown in liquid shaken cultures for 24 hours at 250 rpm. Then equal amounts of mycelia were homogeneously spread onto solid GMM, GMM supplemented with 200 mM KCl, or GMM supplemented with 10 mM CaCl_2_ (time point - 0 h) and further incubated at 37°C. B) and C) Expression levels of *nsdD* and *steA*, respectively, analyzed by qRT-PCR. Total RNA was extracted using Trizol®, from mycelial samples collected at 24 and 48 hours after the shift. The values were normalized to the wild-type (grown on GMM -24 h) levels considered as 100. The error bar represents standard error. Values are the means of three replicates.

Deletion of *sltA* affected early stages of sexual development. A reduction in Hülle cell production was observed in the *sltA* deletion mutant (RSS1.6P) compared to the control (FGSC4) 24 and 48 hours after the shift from liquid to solid GMM (Figure 5A). Addition of K^+^ had no positive effect but calcium partially recovered Hülle cell production in the mutant background (Figure 5A).

### Normal expression of *nsdD* and *steA* requires a *sltA* wild-type allele

We examined whether this defect in the first stages of sexual development was caused by changes in the expression of *nsdD* and *steA* genes, necessary for sexual development in *A. nidulans* [33,34]. *nsdD* expression was reduced in the ∆*sltA* strain (RSS1.6P) as compared to the corresponding control strain (Figure 5B). Supplementation with K^+^ resulted in an additional delay in *nsdD* expression in the *sltA* deletion mutant. Furthermore, *steA* was also reduced in the ∆*sltA* strain as compared to the wild type, at both time points analyzed (Figure 5C). Addition of 10 mM Ca^2+^ resulted in a notable increase in *nsdD* and *steA* transcript levels, particularly in the *sltA* mutant strain, at both time points, 24 and 48 hours respectively (Figure 5B and 5C).

### Expression of *sltA* during different developmental stages

**Figure 6:**
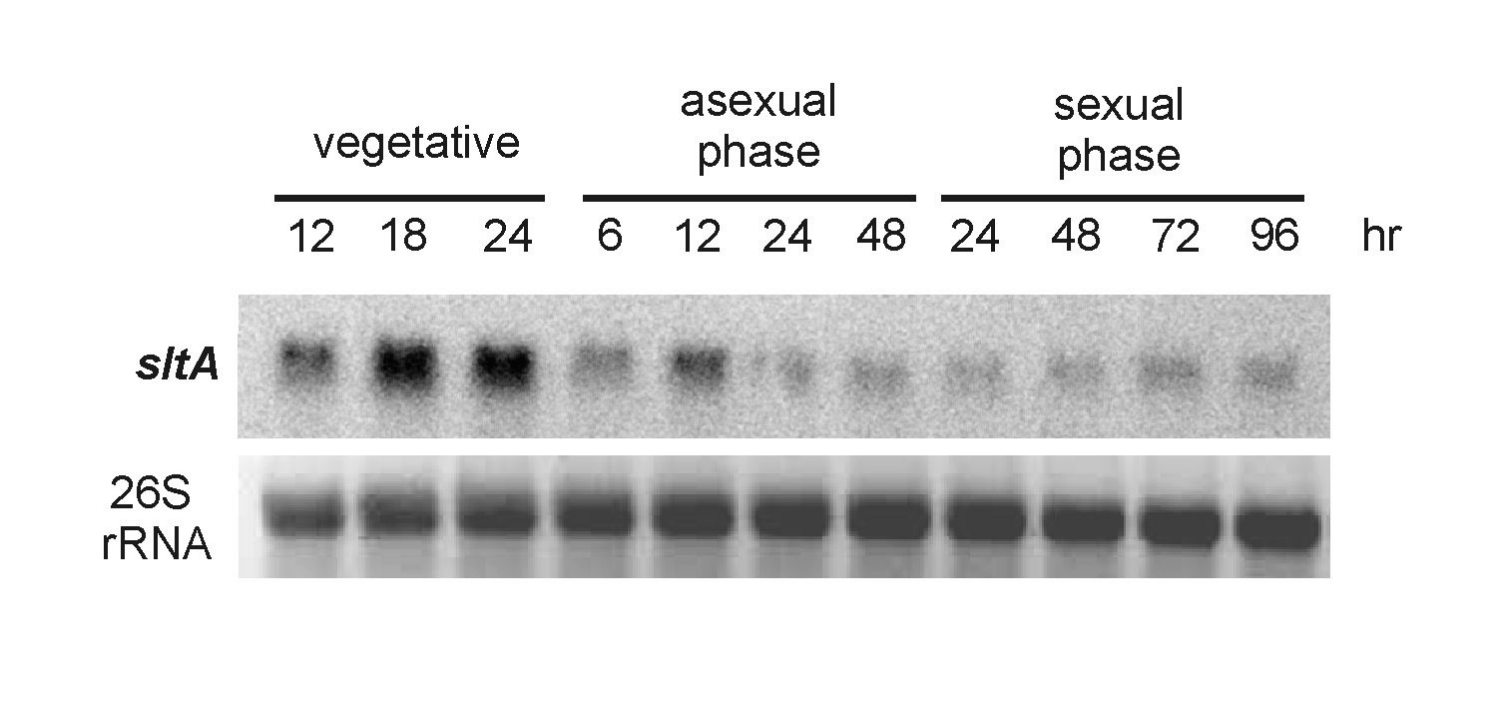
Expression profile of *sltA* during vegetative growth, asexual and sexual-developmental phases in a wild-type strain. A wild-type *velvet* strain (*veA* ^+^, strain WIM126) was cultured in supplemented liquid GMM for vegetative mycelial growth. Samples were collected at the times indicated on top of the figure. For induction of asexual or sexual development, vegetative mycelia were collected by filtration after 24 hours of culture in liquid GMM and placed on top of solid GMM. Samples were collected at the time points indicated on top of the figure. Extracted total RNAs were probed with a radioactively labeled *sltA* coding region. The 26S ribosomal RNA is shown as loading control.

Due to the observed effect of *sltA* deletion on the expression of developmental genes regulating both asexual and sexual morphological differentiation, we also analyzed the expression of *sltA* in the wild type during vegetative growth and also during different developmental stages. Our Northern blot analysis indicated that *sltA* was expressed during vegetative growth as well as during asexual and sexual differentiation at each time point analyzed (Figure 6), with the greatest *sltA* expression during vegetative growth.

### Role of *sltA* in *A. nidulans* ST production

**Figure 7:**
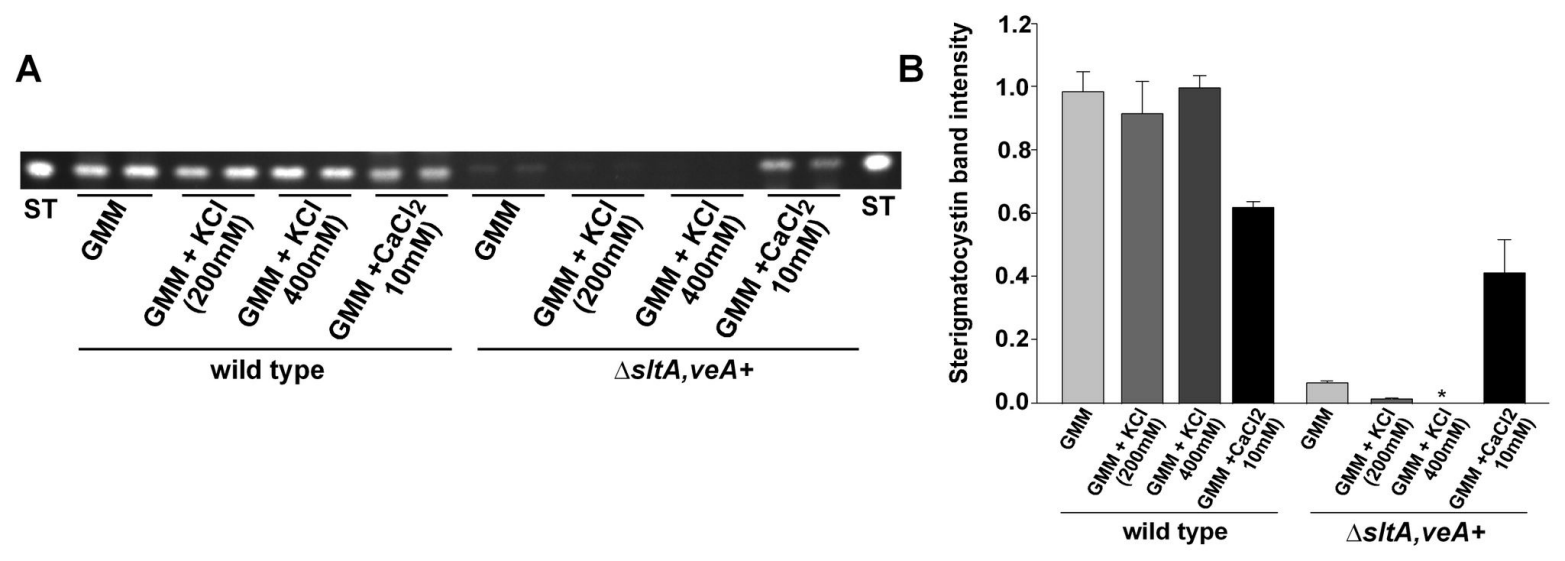
SltA is necessary for normal ST biosynthesis particularly in the presence of potassium while calcium sustains toxin production in the absence of SltA. A) Wild type (FGSC4) and ∆*sltA, veA* ^+^ (RSS1.6P) strains were top-agar inoculated with 5 x 10^6^ conidia per plate on GMM, GMM supplemented with KCl (200 and 400 mM) or CaCl_2_ (10 mM), followed by incubation at 37°C for 5 days. ST toxin was extracted and analyzed by thin layer chromatography (TLC) as described in the experimental procedure section. B) Densitometry displaying the intensity of the ST bands. The densitometry was carried out using the Scion Image 4.03 software. Values are the means of two replicates. The error bar represents standard error.

Mycotoxin analysis indicated that the production of ST is drastically reduced in the *sltA* deletion strains (RSS1.6P and HHF27B) compared to the control strains (FGSC4 and HHF27A) in the *veA* ^+^ or *veA1* genetic background, respectively (Figure 7 and Figure S3), particularly in the presence of 200 mM or 400 mM KCl. Interestingly, ST biosynthesis was partially recovered in the *sltA* deletion strain cultures containing GMM supplemented with 10 mM CaCl_2_ (Figure 7).

### Expression of ST genes is *sltA*-dependent

**Figure 8:**
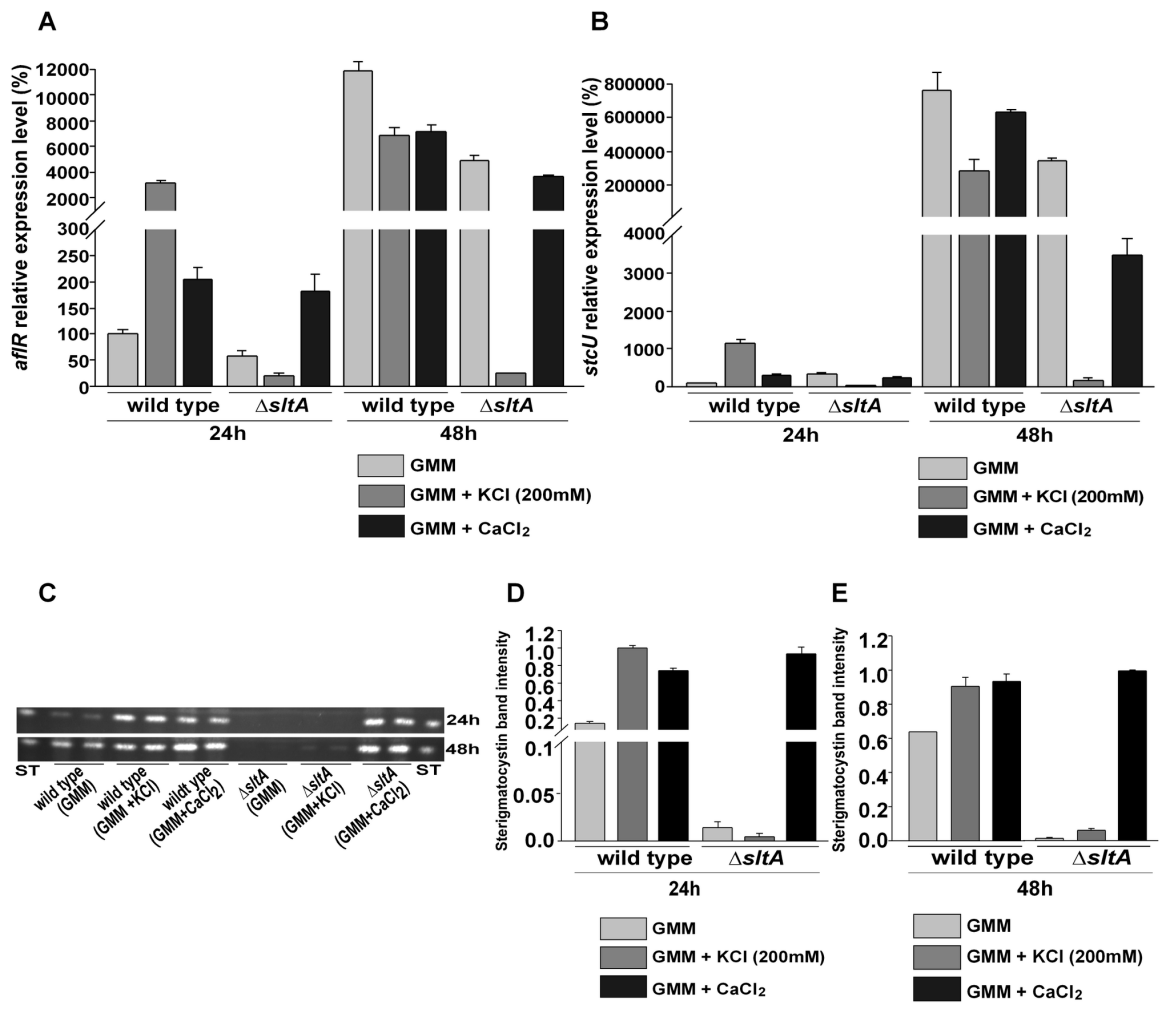
SltA affects *aflR* and *stcU* expression. A) Graph representing the relative expression levels of *aflR* analyzed by qRT-PCR. Strains were grown in liquid GMM, GMM supplemented with 200 mM KCl or 10 mM CaCl_2_ in a shaker incubator for 24 and 48 hours respectively at 250 rpm at 37°C. Total RNA was extracted using Trizol®, from mycelial samples collected at 24 and 48 hours from the liquid cultures. The values were normalized to the wild-type (grown on GMM -24h) levels considered as 100. B) Expression analysis of *steA* by qRT-PCR. The values were normalized to the wild type (grown on GMM -24 h) levels considered as 100. C) TLC analysis of ST produced in wild-type (FGSC4) and ∆*sltA, veA* ^+^ (RSS1.6P) cultures under the same experimental conditions. D) & E) Densitometry displaying the relative intensity of ST bands. Values are the means of two replicates. The error bar represents standard error.

Expression of *aflR*, encoding a transcription factor necessary for the activation of ST gene cluster, and *stcU*, a structural gene in the cluster commonly used as indicator of ST cluster activation, were slightly reduced in the *sltA* deletion strain (RSS1.6P) compared to the wild-type control (Figure 8A and 8B). Potassium acted positively on the expression of these mycotoxin genes in the wild type strain, whereas had a strong negative effect in the null *sltA* mutant (Figure 8A and 8B). Mycotoxin analysis performed under the same experimental conditions also revealed a drastic decrease in ST production in ∆*sltA* with respect to the control (Figure 8C, 8D and 8E). Addition of calcium resulted in increased levels of *aflR* and *stcU* expression as well as ST production in ∆*sltA*.

### Complementation of *∆sltA* with a *sltA* wild-type allele remediated the defects in growth, morphogenesis and mycotoxin production

When a genomic wild-type *sltA* copy was transformed in ∆*sltA, veA* ^+^ (RSS1.6P) the resulting complementation strain recovered wild-type phenotype (FGSC4 phenotype) for growth, asexual and sexual development and production of ST toxin (Figure S4), in addition to tolerance of high concentrations of divalent or monovalent cations (data not shown). ∆*sltA, veA1* (HHF27B) was also complemented for loss of *sltA* function using a similar approach. All mutant phenotypes described to be associated with the absence of *sltA* in this work and in previous work [4] were suppressed (data not shown).

### Promoter analysis

Analysis of the promoter region, consisting of about 3.5 kb upstream of the developmental genes *brlA*, *nsdD* and *steA* and mycotoxin, sterigmatocystin regulatory gene *aflR* revealed the presence of several putative SltA (5’-AGGCA- 3’) binding sites: *brlA* (five), *nsdD* (one), *steA* (three) and *aflR* (five) (Figure S5). Analysis of the complementary strand of each of these promoter regions also revealed additional putative 5’-AGGCA- 3’ (SltA) target sites: *brlA* (three), *nsdD* (five), *steA* (four) and *aflR* (three) (Figure S5).

## Discussion

In this study we have described the role of SltA, the transcription factor mediating cation stress response, on growth, morphological development (asexual and sexual) and ST production in the model fungus *A. nidulans*. SltA has a role in preventing the toxicity of cations such as Na^+^ and K^+^ but not by Ca^2+^; these studies were carried out in strains with *veA1* allele [2–4,10], a mutant allele commonly present in many strains used in *A. nidulans* research laboratories which affects fungal development. In this study SltA function was characterized in *veA*+ (wild type) as well as in *veA1* genetic background.

Our results have revealed a reduction in colony growth and conidial production in the *A. nidulans sltA* deletion mutant, in both *veA1* or *veA* ^+^ genetic backgrounds, indicating that *sltA* is important in fungal growth independently of the type of *veA* allele. The supplementation of potassium resulted in a minimal difference in colony growth of the *sltA* deletion mutant, already very reduced even in the absence of this excess of cation in nitrate-GMM. This medium has been used for decades in genetic studies of both development and mycotoxin production [16,25,26,30–32]. Previous work by Spielvogel et al., 2008 [4] on *sltA* mutant in *veA1* genetic background presented slightly reduced colony growth when grown on Cove’s minimal medium with ammonium tartrate as nitrogen source. However this medium is not suitable to mycotoxin production in *A. nidulans* [35]. These differences in medium composition account for the variations observed in the growth of the *sltA, veA1* mutant in both studies ( [4] and Figure S1). It is possible that a connection between nitrogen metabolism and the role of SltA could exist in *A. nidulans*.

Our study also showed that the reduction in conidiation was accompanied by a reduction in the expression of *brlA*, a key regulator of asexual development [11], in the ∆*sltA* as compared to the wild type. The presence of K^+^ had an opposite effect in the wild type versus the *sltA* ^*-*^ mutant. Potassium elevated *brlA* expression and conidiation in the wild type while it caused a strong decrease in the absence of SltA. This indicates that SltA is not only necessary for normal colony growth but also required for wild-type expression of *brlA* and concomitant conidiation in *A. nidulans*, particularly under potassium stress conditions.

Promoter analysis of *brlA* showed the presence of eight 5’-AGGCA-3’ consensus SltA binding sites (Figure S5). These are distributed within the 2.3 kb region upstream from the *brlA*α translation start site. Interestingly, sites located at -2275 and -2300, the furthermost upstream sites, are in a region that also contains experimentally demonstrated FlbB and FlbD binding sites [36]. The abundance of SltA binding sites together with the fact that this region is subjected to regulation by other DNA binding proteins strongly suggests these putative SltA sites are functional.

In this study we showed that *sltA* transcripts are present during asexual and sexual stages, and demonstrated that sexual development is also impaired in the absence of *sltA*, coinciding with lower expression of *nsdD* and *steA*, known to be necessary for sexual development [33,34]. *nsdD* and *steA* promoter regions also presented putative SltA binding sites. Both sexual and asexual developmental programs were partially recovered when ∆*sltA* cultures were supplemented with calcium, as well as an overall recovery of fungal growth. In *Penicillium* *urticae* , calcium was shown to increase conidiation [37]. Addition of low amounts of calcium to the medium, also led to increased accumulation of *brlA* transcripts in the *sltA* deletion strain. Similarly, partial recovery of Hülle cells, nursing cells for cleistothecial formation [13] coincided with a recovery of *nsdD* and *steA* transcript levels, which are abnormally low in ∆*sltA* cultures.

Previous reports showed that calcium alleviates a number of defects caused by the null *sltA* in early growth stages. For instance, *sltA* ^-^ strains present altered number, size and distribution of vacuoles, and calcium remediates this phenotype [10]. Abnormal expression of calcium vacuolar ATPase coding genes, *pmcA* and *pmcB*, were observed in ∆*sltA* background and such phenotype was associated with a cytoplasmic Ca^2+^ depletion. This effect of "calcium auxotrophy was caused by the combination of ∆*sltA* with a null allele of *halA*, a kinase involved in cation stress signaling [10,38]. Findon et al. [10] also showed the link between alkaline cation uptake systems and calcium through the isolation of suppressors of "calcium auxotrophy" phenotype, of which several mutations were isolated in a Na^+^/H^+^ transporter, NhaA, and in a K^+^ transporter, TrkB [10]. Additionally a role of SltA in intracellular traffic of endomembranes has been proposed as a result of isolation of loss-of-function mutations in *sltA* as extragenic suppressors of the lethal phenotype caused by null alleles of certain vacuolar-protein-sorting (vps) genes. An interpretation of this genetic effect of *sltA* mutations was an altered plasma-membrane trafficking of small vesicles derived from extreme mutations in these *vps* genes [39]. It is possible that the remediation of colonial growth, asexual and sexual development defects observed in the *sltA* deletion mutant supplemented with calcium could be the result of a restoration of intracellular membrane-based trafficking and/or signaling systems. Previously, it was also shown that high gradient of Ca^2+^ is necessary for tip hyphal extension in filamentous fungi [40]. Decreasing the Ca^2+^ influx leads to reduction of hyphal extension and increased branching, while high extracellular calcium inhibits branching and induces hyphal elongation [41]. Calmodulin is an essential signaling element that senses intracellular calcium and accumulates at the hyphal tip in *A. nidulans* [42]. To date a relationship between CaM and SltA has not been investigated but the possible effect of SltA on the regulation of free cytosolic calcium might alter the signaling cascade depending on calmodulin-Ca^2+^ complex, affecting, among other cellular processes, the calcium gradient that promotes hyphal tip elongation.

Although a positive effect of calcium in conidiation has been also reported in *Penicilli* species [43,44], this is, to our knowledge, the first report showing this Ca^2+^-dependent conidiation increase in *Aspergillus* . In our studies this increase in asexual development was accompanied by a remarkable increase in *brlA* expression. Calcium signaling and *brlA* expression has been connected through studies on the transcription factor CrzA in *Aspergillus fumigatus*, and the activity of the protein phosphatase calcineurin which has been reported to have a role in conidiation and sexual development [45].

A regulatory link between development and secondary metabolism has been experimentally demonstrated in filamentous fungi, including the model fungus *A. nidulans* [14,16,17,46]. For this reason we investigated the role of *sltA* in mycotoxin production. Under standard culture conditions ∆*sltA* showed a decrease in ST biosynthesis compared to the wild type. This decrease was even more notable under potassium stress either in *veA1* or *veA* ^+^ genetic backgrounds, coinciding with delayed expression of *aflR* and *stcU*. This indicates that *sltA* influences ST by controlling *aflR* and consequently the activation of the ST gene cluster. Putative SltA binding sites are also present in the *aflR* promoter region. Addition of calcium not only partially rescued the morphological defects in the *sltA* mutant, but also resulted in a recovery in the levels of ST biosynthesis, parallel to a recovery of *aflR* and *stcU* expression levels. Additionally to the described effect of *sltA* deletion on the expression of ST genes, possible alterations in vesicle trafficking [39] could also contribute to the observed decrease in mycotoxin biosynthesis [48].

Developmental defects and a reduction of ST production in ∆*sltA* supports the role of SltA in normal growth, conidiation, sexual development and mycotoxin production in *A. nidulans*, suggesting that maintaining homeostasis and proper response to changes in the concentration of cations are necessary to allow several cellular functions including morphogenesis and secondary metabolism. These results contribute to a better understanding of the roles associated with cation regulation and signaling mechanisms in filamentous fungi, particularly in the genus *Aspergillus* .

## Supporting Information

Figure S1 Effect of medium on *veA1*∆*sltA* and *veA*+∆*sltA* colony growth. ∆*sltAveA1* (HHF27B), ∆*sltAveA* ^+^ (RSS1.6P) and corresponding control strains (HHF27A and FGSC4 respectively) were point-inoculated on plates containing GMM as described by Käfer [28], or GMM supplemented with 200 mM, containing sodium nitrate as nitrogen source (A, B), or on minimum medium as described by Cove [10,47], or Cove medium plus 200 mM, containing ammonium tartrate as nitrogen source (C, D). Cultures were incubated at 37°C for 5 days.
(TIF)

Figure S2 SltA is necessary for normal growth and conidiation also in a *veA1* genetic background. A) Control strain (HHF27A) and ∆*sltA*, *veA1* (HHF27B) point-inoculated cultures containing GMM, or GMM supplemented with 200 mM or 400 mM KCl were incubated at 37°C for 5 days. B) & C) Measurement of the radial colony growth. D) & E) Quantification of conidial production from top-agar inoculated cultures (5 x 10^6^ spores/plate). Values are means of three replicates. The error bar indicates standard error.
(TIF)

Figure S3 Effect of *sltA* deletion on ST biosynthesis was similar in both *veA* ^+^ and *veA1* genetic backgrounds. A) & C) TLC analysis of ST produced by the wild-type and ∆*sltA, veA* ^+^ strains on GMM or GMM supplemented with KCl (200 and 400 mM). Strains were top-inoculated with 5 x 10^6^conidia per plate and incubated at 37°C for 5 days. ST was extracted and analyzed as described in the experimental procedure section. B) & D) Densitometry displaying the intensity of the ST bands in A and C respectively. E) & G) TLC analysis of ST produced by the HHF27B control and ∆*sltA, veA1* strains on GMM or GMM supplemented with KCl (200 and 400 mM). The experiment was carried out as above. F) and H) Densitometry displaying the intensity of the ST bands in E and G respectively. Densitometries were carried out using the Scion Image 4.03 software. Values are the means of three replicates. The error bar represents standard error.
(TIF)

Figure S4 **Complementation of** ∆***sltA* with a *sltA* wild-type allele rescues wild-type phenotype**.
A) For reconstitution of null *sltA* into a wild-type *sltA* locus, a PCR fragment amplified using primer pair sltA_F and sltA_R containing the wild-type *sltA* genomic sequence between coordinates 2396219 and 2400452 of chromosome VI (http://www.aspgd.org/) was used for transformation of ∆*sltA, veA* ^+^ prototroph (RSS1.6P). Positive transformants recovering SltA activity were selected onto glucose minimal medium containing 1M sucrose as osmotic stabilizer and 0.3 M LiCl. Presence of a wild-type genomic copy of *sltA* was verified by PCR techniques. Agarose electrophoresis of PCR products using oligonucleotides sltA47 and sltA39. Lanes contain PCR products using as template genomic DNAs from RSS1.6P (lane 1), FGSC4 (lane 2), HHF27B (lane 3), reconstituted *sltA* transformants in RSS1.6P (lanes 4-9), a reconstituted *sltA* transformant in HHF27B (lane 10). Lanes A and B are control PCRs for negative amplification of *sltA* locus using HHF27A genomic DNA. Absence of mutations in the coding region of *sltA* was verified by sequencing. Mw is DNA molecular maker VII (Roche). Complementation of ∆*sltA, veA* ^+^ rescues wild-type conidiation and cleistothecial production in *A. nidulans* (B), as well as ST biosynthesis (C). Plates in (B) were top-agar inoculated with 5 x10^6^ spores of wild type (FGSC4), ∆*sltA, veA*+ (RSS1.6P), and complementation strain (RSS1.6P-com) on GMM or GMM plus 200 mM KCl, and incubated for 5 days. ST shown in (C) was extracted from liquid shaken cultures grown for 4 days.
(TIF)

Figure S5 In-silico analysis of putative SltA transcription factor binding sites in the promoter regions. A) *brlA* B) *nsdD* C) *steA* and D) *aflR*. Numbers indicate the positions of the putative SltA binding site upstream to the ATG start. Green solid lines (5’–3’) indicate the putative SltA binding sites identified in the 3.5 kb region upstream of the above mentioned genes, while blue solid lines indicate the SltA binding sites on the complementary strand (3’–5’) of the promoter region. Coordinates at both ends of the promoter regions are with respect to the position in the contig. Yellow boxes indicate CDS and white boxes indicate exons of 5' and 3' UTRs present in the genomic regions under analyses. In the case of *brlA*, ATG of *brlA* alpha transcript is shown but also the position of *brlA* beta initiation codon and first exon.
(TIF)
