# Supplementary figures and images for: Role of the Zinc Finger Transcription Factor SltA in Morphogenesis and Sterigmatocystin Biosynthesis in the Fungus Aspergillus nidulans
Source: PLoS One. 2013 Jul 1;8(7):e68492. doi: 10.1371/journal.pone.0068492 (PMC3698166; doi:10.1371/journal.pone.0068492)

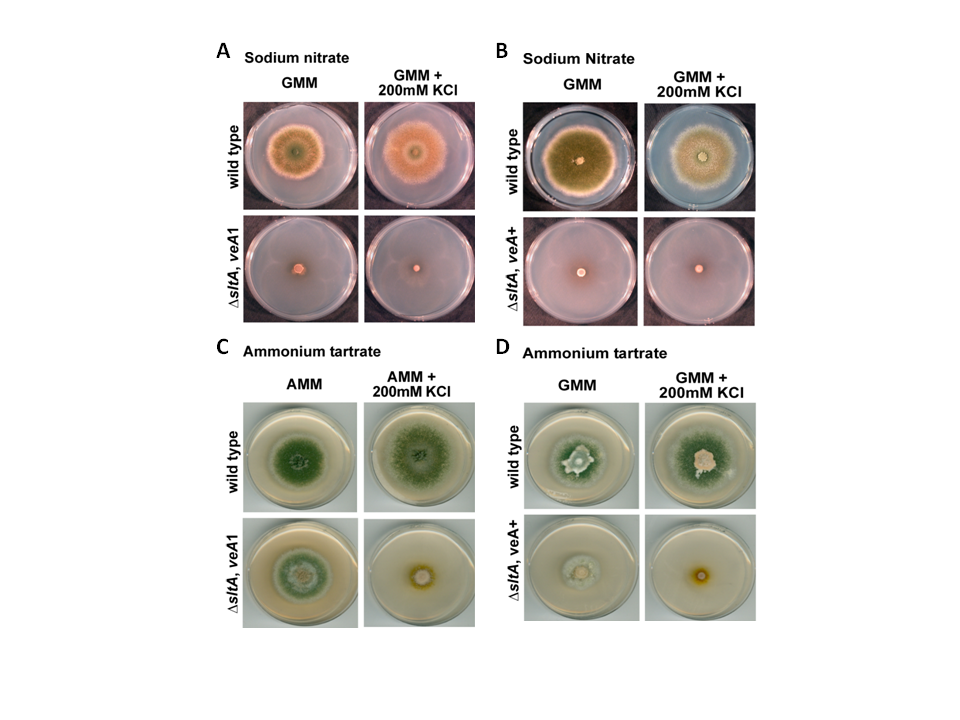

Supplement: Figure S1 — ∆sltAveA1 (HHF27B), ∆sltAveA + (RSS1.6P) and corresponding control strains (HHF27A and FGSC4 respectively) were point-inoculated on plates containing GMM as described by Käfer [28], or GMM supplemented with 200 mM, containing sodium nitrate as nitrogen source (A, B), or on minimum medium as described by Cove [10,47], or Cove medium plus 200 mM, containing ammonium tartrate as nitrogen source (C, D). Cultures were incubated at 37°C for 5 days. (TIF) [file pone.0068492.s001.tif]

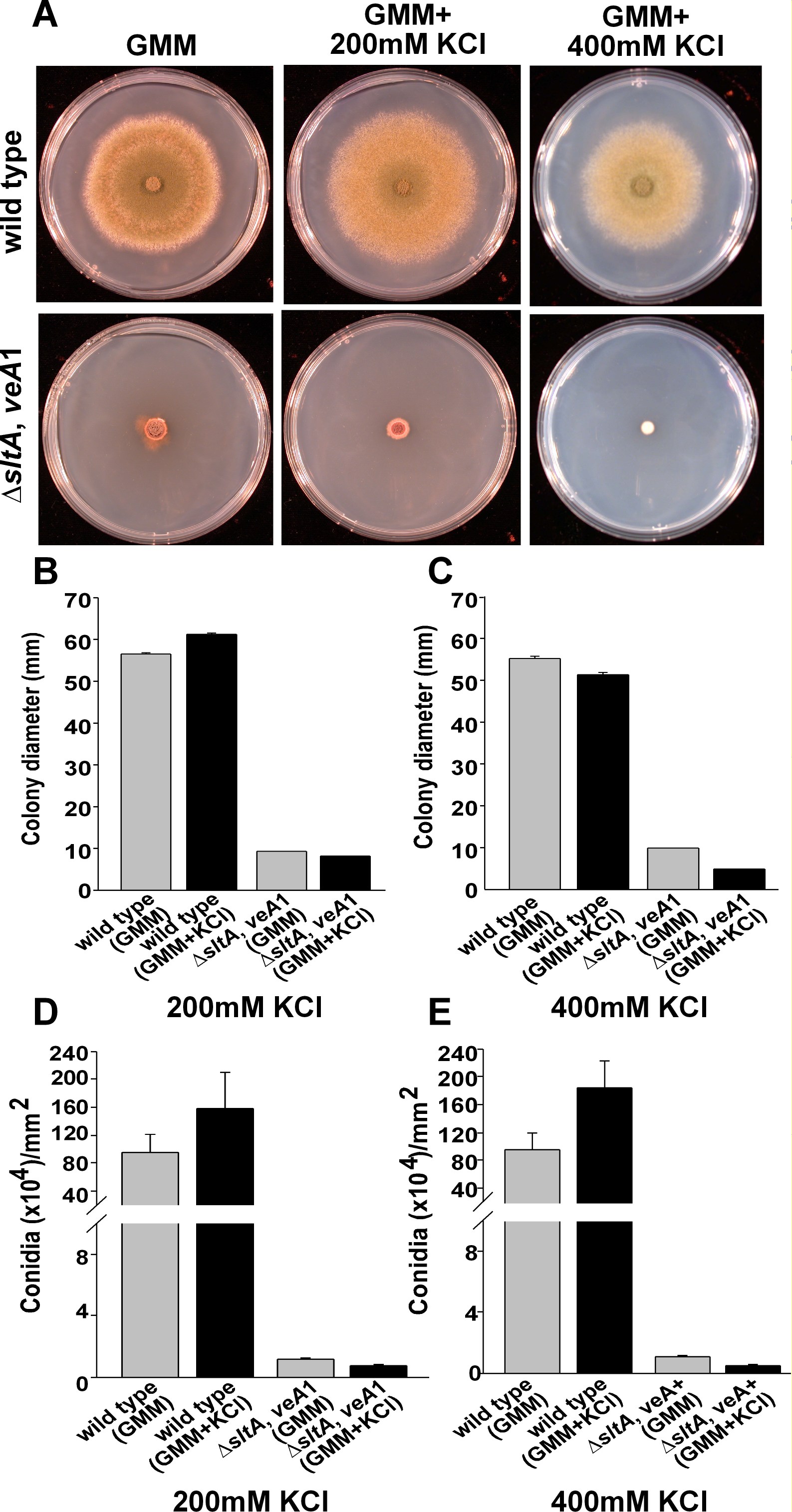

Supplement: Figure S2 — A) Control strain (HHF27A) and ∆sltA, veA1 (HHF27B) point-inoculated cultures containing GMM, or GMM supplemented with 200 mM or 400 mM KCl were incubated at 37°C for 5 days. B) & C) Measurement of the radial colony growth. D) & E) Quantification of conidial production from top-agar inoculated cultures (5 x 10^6^ spores/plate). Values are means of three replicates. The error bar indicates standard error. (TIF) [file pone.0068492.s002.tif]

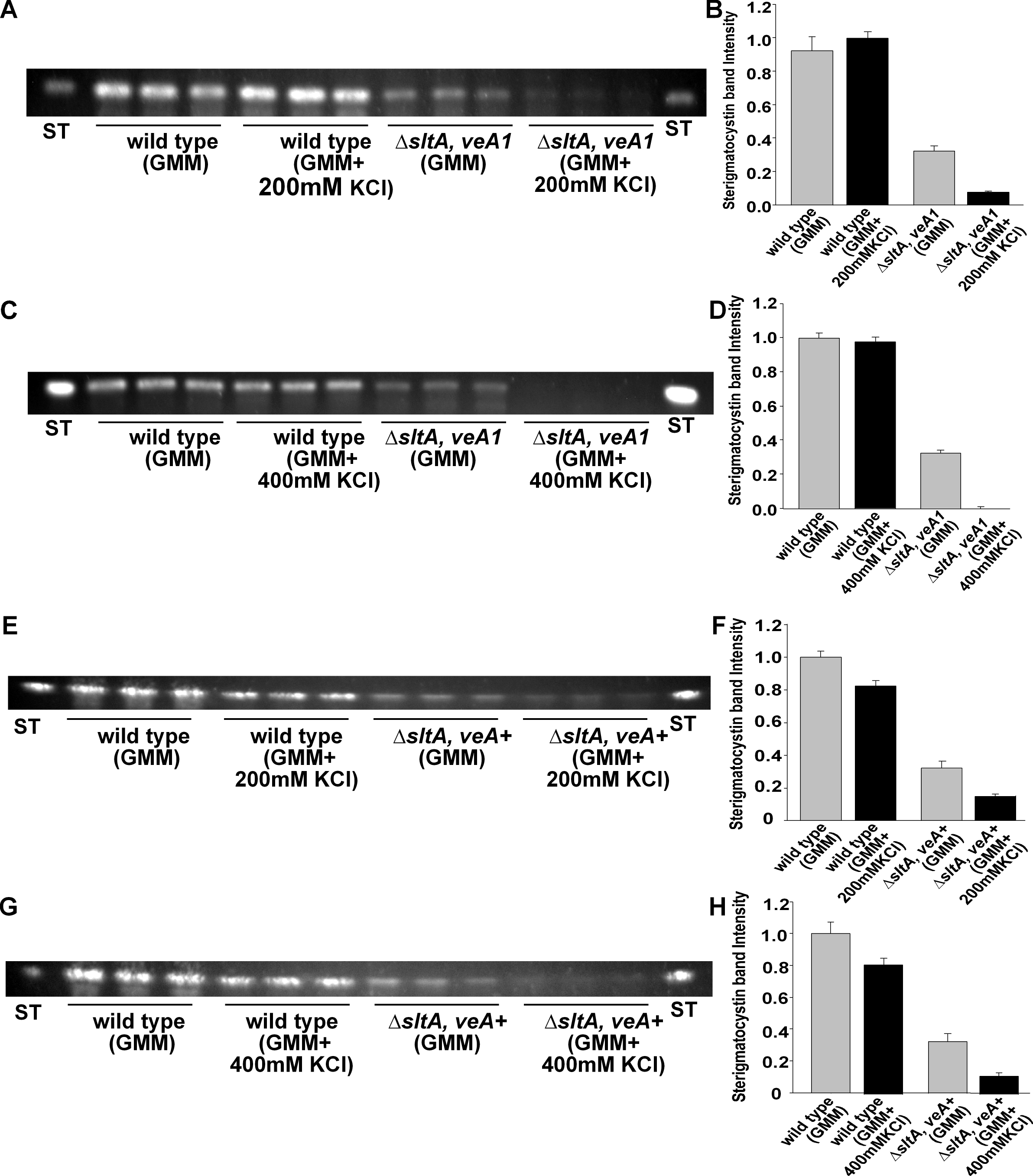

Supplement: Figure S3 — A) & C) TLC analysis of ST produced by the wild-type and ∆sltA, veA + strains on GMM or GMM supplemented with KCl (200 and 400 mM). Strains were top-inoculated with 5 x 10^6^conidia per plate and incubated at 37°C for 5 days. ST was extracted and analyzed as described in the experimental procedure section. B) & D) Densitometry displaying the intensity of the ST bands in A and C respectively. E) & G) TLC analysis of ST produced by the HHF27B control and ∆sltA, veA1 strains on GMM or GMM supplemented with KCl (200 and 400 mM). The experiment was carried out as above. F) and H) Densitometry displaying the intensity of the ST bands in E and G respectively. Densitometries were carried out using the Scion Image 4.03 software. Values are the means of three replicates. The error bar represents standard error. (TIF) [file pone.0068492.s003.tif]

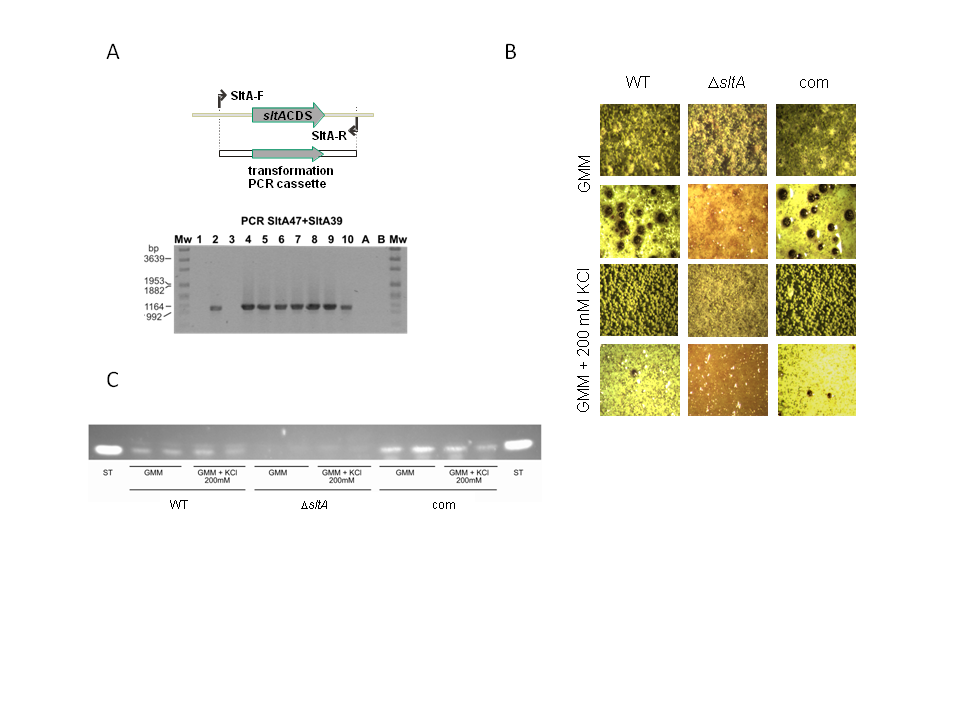

Supplement: Figure S4 — Complementation of ∆sltA with a sltA wild-type allele rescues wild-type phenotype. A) For reconstitution of null sltA into a wild-type sltA locus, a PCR fragment amplified using primer pair sltA_F and sltA_R containing the wild-type sltA genomic sequence between coordinates 2396219 and 2400452 of chromosome VI (http://www.aspgd.org/) was used for transformation of ∆sltA, veA + prototroph (RSS1.6P). Positive transformants recovering SltA activity were selected onto glucose minimal medium containing 1M sucrose as osmotic stabilizer and 0.3 M LiCl. Presence of a wild-type genomic copy of sltA was verified by PCR techniques. Agarose electrophoresis of PCR products using oligonucleotides sltA47 and sltA39. Lanes contain PCR products using as template genomic DNAs from RSS1.6P (lane 1), FGSC4 (lane 2), HHF27B (lane 3), reconstituted sltA transformants in RSS1.6P (lanes 4-9), a reconstituted sltA transformant in HHF27B (lane 10). Lanes A and B are control PCRs for negative amplification of sltA locus using HHF27A genomic DNA. Absence of mutations in the coding region of sltA was verified by sequencing. Mw is DNA molecular maker VII (Roche). Complementation of ∆sltA, veA + rescues wild-type conidiation and cleistothecial production in A. nidulans (B), as well as ST biosynthesis (C). Plates in (B) were top-agar inoculated with 5 x10^6^ spores of wild type (FGSC4), ∆sltA, veA+ (RSS1.6P), and complementation strain (RSS1.6P-com) on GMM or GMM plus 200 mM KCl, and incubated for 5 days. ST shown in (C) was extracted from liquid shaken cultures grown for 4 days. (TIF) [file pone.0068492.s004.tif]

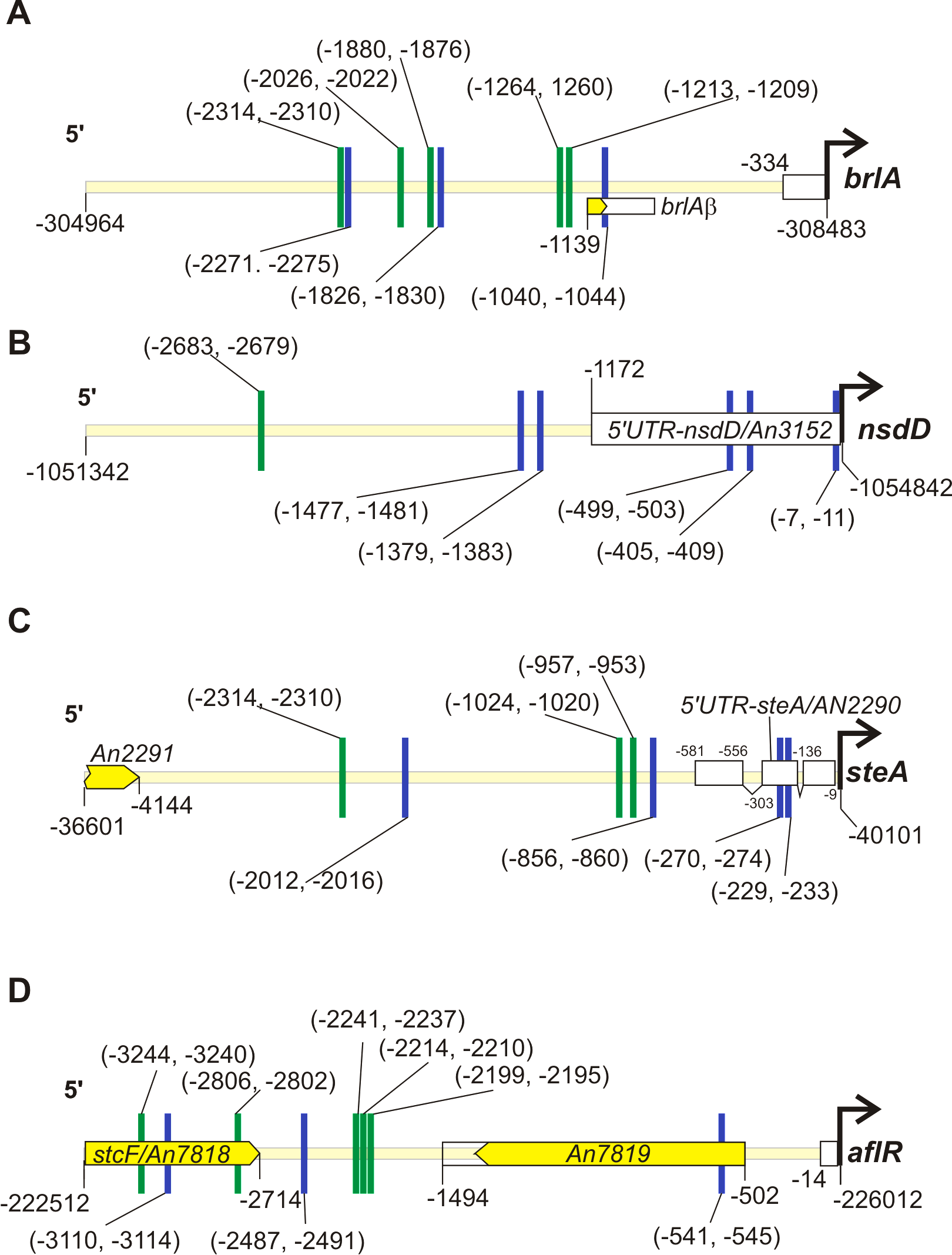

Supplement: Figure S5 — A) brlA B) nsdD C) steA and D) aflR. Numbers indicate the positions of the putative SltA binding site upstream to the ATG start. Green solid lines (5’–3’) indicate the putative SltA binding sites identified in the 3.5 kb region upstream of the above mentioned genes, while blue solid lines indicate the SltA binding sites on the complementary strand (3’–5’) of the promoter region. Coordinates at both ends of the promoter regions are with respect to the position in the contig. Yellow boxes indicate CDS and white boxes indicate exons of 5' and 3' UTRs present in the genomic regions under analyses. In the case of brlA, ATG of brlA alpha transcript is shown but also the position of brlA beta initiation codon and first exon. (TIF) [file pone.0068492.s005.tif]
